# Obituary

**Published:** 1916-12

**Authors:** 


### OBITUARY

GORDON WHITE, D. D. S. Dr. Gordon White, of Nashville, Tenn., died at his home in Nashville, December 6th, in his sixtieth year. He graduated from the Baltimore Dental College in 1879, and has been in active practice in Nashville since, and almost to the day of his death. Few men have been held in such high esteem by his professional confreres, and none have been better served than his patients. He was a thoughtful and ardent student of his profession and was well known as a man of the highest attainments, nationally as well as in his own country. He was also a man of strong personality, and was a leader in professional and social affairs. He has been markedly honored by the profession of his city, his state, in the South, and in fact in the professional activities of the entire nation. His practice was not local, but he had patients referred to him from distant cities and states. His skill and agreeable personality attached his patients by the strongest ties of confidence and affection. He treated pathologic conditions of the mouth most successfully. His career was a brilliant one, and his death will be a severe loss to the profession of the South.

DR. NORMAN G. ROECH, born at Phoenix, Rhode Island, October 20, 1879, and died at Winthrop, Massachusetts, May 25, 1916.

Whereas, our beloved Fellow, Dr. Norman G. Roech, has been removed from us by Divine Providence, it is fitting that we should make a record of his death and express our sorrow over the untimely close of a successful career; and

Whereas, by his genial disposition, his skill as a practitioner of his chosen specialty, he has been a credit to the dental profession; therefore, be it

Resolved, that we, the Fellows of the American Academy of Dental Science, feeling deeply the loss we have sustained, hereby express our appreciation of his friendship, and our sorrow at his death, and be it further

Resolved, that these resolutions be spread upon our minutes and that a copy be sent to the widow and his family.

JOHN W. FORBES,
FRANK T. TAYLOR,
LAWRENCE W. BAKER.

DR. CHEEVER, who died in Boston, December 27, 1915, was an Honorary Fellow of the American Academy. He was a man of sterling character, and a leader in his profession, very prominent in the medical and surgical world. He died just entering his eighty-fifth year. He was a broad and noble minded man, having an abundant love for his profession. He not only worked faithfully as a great surgeon and teacher, but also lifted his associates to high and still higher aims.

He was born in Portsmouth, New Hampshire, November 30, 1831. His father and grandfather were physicians. He served the Harvard Medical School for thirty-three years as teacher and professor. He was connected with the Boston City Hospital for fifty-two years. He saw service in the Civil War. He was a writer of marked ability, writing for the *Atlantic Monthly* and the *North American Review*, also a writer for all the leading medical and surgical journals in this country. He was known and esteemed for his charities, was a public spirited citizen and an excellent business man. As an example, advisor and friend, he was all that could be desired,—faithful, loyal, reliable, under all circumstances. All honor to the academy for honoring such a man. His loss to the entire surgical world is a deep and lasting loss.

His life and death, his beauty of character, remind one of the closing lines of Bryant's greatest poem.

"So live, that when thy summons comes to join
The innumberable caravan that moves

To the pale realms of shade, where each shall take
His chamber in the silent halls of death,
Thou go not like the quarry-slave at night,
Scourged to his dungeon; but, sustained and soothed
By an unfaltering trust, approach thy grave,
Like one who wraps the drapery of his couch
About him, and lies down to pleasant dreams."

Resolved, That the American Academy of Dental Science has lost one of its most esteemed Honorary Fellows, and his profession, a noble leader. His memory will be revered by all who love high character, noble service and the sterling attributes of the finest type of man.

Respectfully submitted,

R. R. ANDREWS,
EDWARD C. BRIGGS,
FORREST G. EDDY.

